# Whole genome sequence analysis reveals high genomic diversity and potential host-driven adaptations among multidrug-resistant *Escherichia coli* from pre-weaned dairy calves

**DOI:** 10.3389/fmicb.2024.1420300

**Published:** 2024-09-03

**Authors:** Katie Y. Lee, Cory L. Schlesener, Sharif S. Aly, Bihua C. Huang, Xunde Li, Edward R. Atwill, Bart C. Weimer

**Affiliations:** ^1^Department of Population Health and Reproduction, School of Veterinary Medicine, University of California, Davis, Davis, CA, United States; ^2^100K Pathogen Genome Project, School of Veterinary Medicine, University of California, Davis, Davis, CA, United States; ^3^Veterinary Medicine Teaching and Research Center, School of Veterinary Medicine, University of California, Davis, Tulare, CA, United States

**Keywords:** antimicrobial resistance (AMR), multidrug resistance (MDR), *Escherichia coli*, dairy calves, calf diarrhea, whole genome sequencing

## Abstract

Food-producing animals such as dairy cattle are potential reservoirs of antimicrobial resistance (AMR), with multidrug-resistant (MDR) organisms such as *Escherichia coli* observed in higher frequency in young calves compared to older cattle. In this study, we characterized the genomes of enteric MDR *E. coli* from pre-weaned dairy calves with and without diarrhea and evaluated the influence of host-level factors on genomic composition. Whole genome sequence comparative analysis of *E. coli* (*n* = 43) revealed substantial genomic diversity that primarily clustered by sequence type and was minimally driven by calf diarrheal disease status (healthy, diarrheic, or recovered), antimicrobial exposure, and dietary zinc supplementation. Diverse AMR genes (ARGs)—including extended-spectrum beta-lactamase genes and quinolone resistance determinants—were identified (*n* = 40), with unique sets of ARGs co-occurring in gene clusters with large AMR plasmids IncA/C2 and IncFIB(AP001918). Zinc supplementation was not significantly associated with the selection of individual ARGs in *E. coli*, however analysis of ARG and metal resistance gene pairs identified positive associations between certain aminoglycoside, beta-lactam, sulfonamide, and trimethoprim ARGs with acid, tellurium and mercury resistance genes. Although *E. coli* in this study lacked the typical virulence factors of diarrheagenic strains, virulence genes overlapping with those in major pathotypes were identified. Among the 103 virulence genes detected, the highest abundance and diversity of genes corresponded to iron acquisition (siderophores and heme uptake). Our findings indicate that the host-level factors evaluated in this study were not key drivers of genomic variability, but that certain accessory genes in enteric MDR *E. coli* may be enriched. Collectively, this work provides insight into the genomic diversity and host-microbe interface of MDR *E. coli* from pre-weaned dairy calves.

## 1 Introduction

*Escherichia coli* is a diverse and ubiquitous organism present in the healthy enteric microbiome of humans and animals and as a pathogen responsible for various diarrheagenic and extraintestinal diseases (Jackson et al., 2011; Braz et al., 2020). The occurrence of antimicrobial resistant (AMR) *E. coli* in food-producing animals, such as dairy cattle, has been identified across various cattle groups in farm environmental matrices, feces, food products (e.g., milk and cheese), and clinical samples (e.g., diarrhea and clinical mastitis) (Ombarak et al., 2018; Formenti et al., 2021; Jeamsripong et al., 2021; Majumder et al., 2021; Imre et al., 2022). The prevalence and persistence of drug-resistant *E. coli* is both a veterinary and human medicine concern, with pathogenic strains compromising animal health and safety of food products, and commensals serving as important reservoirs for the dissemination of AMR.

Multidrug-resistant (MDR) *E. coli* have been observed in higher frequency in younger cattle, particularly in calves around 2 weeks in age (Berge et al., 2005, 2010). This age-dependent and transient increase in AMR of dairy calves is thought to be driven by the early developing gut microbiome, in which initial exposure to the environment, antibiotic therapy, dietary changes, and other factors collectively contribute to the rapid establishment of the bovine resistome (Khachatryan et al., 2004; Noyes et al., 2016; Liu et al., 2019; Springer et al., 2019; Oh et al., 2020). Previous studies have demonstrated the dynamic nature of AMR selection and enrichment in calves, with the acquisition of AMR occurring beyond influences of antibiotic exposure (Liu et al., 2019; Haley and Van Kessel, 2022) and calves harboring greater diversity in AMR than the potential sources (e.g., dam) seeding their resistome (Haley et al., 2020; Massé et al., 2021). Additionally, studies have suggested that biocides used as disinfectants and heavy metal additives in feed may contribute to the co-selection of AMR with biocide and metal resistance (Wales and Davies, 2015; Cheng et al., 2019).

In pre-weaned dairy calves, diarrhea is the leading cause of morbidity and mortality, which frequently results in antimicrobial treatment (Berchtold and Constable, 2009; Habing et al., 2017). To reduce AMR without compromising animal health, antimicrobial alternatives such as dietary zinc supplementation have been explored and shown to be effective in preventing diarrhea and expediting diarrheal recovery (Glover et al., 2013; Feldmann et al., 2019; Chang et al., 2020; Ma et al., 2020; Wo et al., 2022). In this work, we evaluated fecal MDR *E. coli* isolates from pre-weaned dairy calves in a zinc supplementation clinical trial using whole genome sequencing (WGS) comparative analysis. The objective of this study was to characterize AMR and virulence genes and to evaluate calf diarrheal disease status, dietary zinc supplementation, and antimicrobial treatment as potential drivers of genomic variability in MDR *E. coli*. We hypothesize that these host-level factors will contribute to differences in genomic AMR, virulence, and metal resistance profiles, and that the presence of certain genes will provide insight into the persistence of enteric MDR *E. coli* in calves.

## 2 Materials and methods

### 2.1 Isolate source

Fecal *E. coli* isolates in this study were obtained from pre-weaned dairy calves enrolled in a double-blind, block-randomized, placebo-controlled zinc supplementation clinical trial assessing dietary zinc supplementation on diarrhea prevention and calf health. Details on the original trial procedures were previously described (Feldmann et al., 2019). Briefly, all calves were under the same management practices (e.g., housing and diet) and standard on-farm treatment protocols. The repository of 43 *E. coli* isolates correspond to pre-weaned dairy calves 2 weeks in age (range: 14–16 days). One representative fecal *E. coli* isolate per calf was used for analysis, with each isolate corresponding to a calf after 14 consecutive days of dietary zinc sulfate or placebo treatment. Treatments were administered during morning milk feeding with calves in the zinc group receiving 0.22 g zinc sulfate monohydrate (80 mg of elemental zinc) (Sigma-Aldrich Company, St. Louis, MO, United States) with 0.44 g milk replacer powder, and calves in the placebo group receiving only 0.44 g milk replacer powder (Feldmann et al., 2019). At the time of isolate collection, calves were in various stages of diarrheal disease (pre-diarrheic/healthy, diarrheic, or recovered) and exposure to antimicrobial treatment for diarrhea (0, 1, or 2 doses of 31.5 mL (1,575 mg) spectinomycin administered once daily, SpectoGard, Bimeda, Inc., Le Sueur, MN, United States). Other antimicrobial exposures included tetracycline and neomycin administered through daily milk, which were consistent in dosage and duration over time for all calves throughout the study. Calf-level data corresponding to isolates were collected from daily assessment records for individual calves. All isolates were confirmed as *E. coli* using conventional PCR and underwent antimicrobial susceptibility testing (AST) using broth microdilution and the NARMS Gram Negative panel (YCMV3AGNF) as previously described (Lee et al., 2024).

### 2.2 DNA extraction and whole genome sequencing (WGS)

Genomic DNA was extracted from pure overnight *E. coli* cultures per manufacturer’s protocol using the Qiagen’s DNeasy Blood and Tissue kit (Qiagen, Valencia, CA, United States). WGS was conducted using methods from the 100 K Pathogen Genome Project as previously described (Weis et al., 2017; Bandoy and Weimer, 2020; Aguilar-Zamora et al., 2022; Hurtado et al., 2022; Woerde et al., 2023; Hernández-Juárez et al., 2021). Briefly, genomic DNA purity and integrity were assessed using the Nanodrop and the Agilent 2200 TapeStation with the Genomic DNA ScreenTape Assay (Agilent Technologies, Inc., Santa Clara, CA, United States), respectively. Sequencing libraries were constructed using the KAPA HyperPlus library preparation kit (Roche Sequencing Solutions, Pleasanton, CA, United States). Double-stranded genomic DNA was fragmented and indexed using Weimer 384 TS-LT DNA barcodes (Integrated DNA Technologies, Coralville, IA, United States), followed by dual-SPRI size selection and PCR amplification. Final library sizes were confirmed on the LabChip GX using the HT DNA 1 K kit (PerkinElmer, Waltham, MA, United States). Library quantification was conducted using the KAPA Library Quantification Kit (Roche Sequencing Solutions, Pleasanton, CA, United States) to ensure normalized concentrations for sequencing pooling. Final libraries were sequenced using the Illumina HiSeq X Ten with PE150.

### 2.3 Whole genome assembly and comparison

Genomic sequence data was processed as previously described (Bandoy and Weimer, 2020; Higdon et al., 2020; Flores-Valdez et al., 2021; Miller et al., 2021; Depenbrock et al., 2024). Briefly, Trimmomatic was used to remove low-quality sequence and adapters, and FastQC was used to review sequence quality. Paired-end reads from WGS were assembled using Shovill with the SPAdes assembler and a Kmer size of 31. Quality of assemblies was then evaluated using CheckM. Genome similarity was measured using Sourmash with Minhash signatures with a Kmer length of 31 and scaled sketch size of 100,000 per megabase (Brown and Irber, 2016). The matrix output from Sourmash was visualized in R using the pheatmap package (RDocumentation, 2024).

### 2.4 Multilocus sequence typing (MLST) and pangenome analysis

The sequence type (ST) for each genome was determined based on the Achtman seven-locus scheme (*adk*, *fumC*, *gyrB*, *icd*, *mdh*, *purA*, and *recA*) using the PubMLST database (Kaas et al., 2012; PubMLST, 2024). Pangenome analysis was conducted using Roary as described previously (Page et al., 2015; Bandoy and Weimer, 2020; Miller et al., 2021). Pangenome composition and gene diversity estimation were then visualized using open source python script ‘roary_plots.py’ and native Rscript (create_pan_genome_plots.R), respectively (Higdon et al., 2020).

### 2.5 Identification of antimicrobial resistance genes (ARGs), virulence genes, metal resistance genes, and plasmid replicons

Genetic determinants for antimicrobial resistance (ARGs), virulence, metal resistance, and plasmid replicons were determined using Abricate and the ResFinder, VFDB, BacMet, and PlasmidFinder databases, respectively (Zankari et al., 2012; Carattoli et al., 2014; Pal et al., 2014; Chen et al., 2016; Seemann, 2024). Additionally, SNP based resistance for quinolones was identified using RGI with the CARD database (Alcock et al., 2020a,b). Hits were determined if meeting the criteria of ≥90% coverage and ≥ 95% identity. For metal resistance genes, only experimentally confirmed genes were included in the analysis.

### 2.6 Data analyses

Descriptive statistics on the distribution of ARGs, virulence factors, metal resistance genes, and plasmid replicons were conducted in SAS OnDemand for Academics. Differences in the mean number of ARGs and virulence genes by factors of treatment group, diarrhea status, and number of therapeutic antibiotic doses were evaluated using a *t*-test/ANOVA or Mann–Whitney U test.

Proportions of *E. coli* genomes with presence of ARGs and virulence factors were plotted as heatmaps in R using the pheatmap package. Rows of the heatmaps were clustered using the Euclidean distance metric and complete linkage method. Bar plots and violin plots of the distribution of ARGs and virulence factors, respectively, were visualized in R using ggplot2 (Wickham et al., 2023).

To investigate the differences in antimicrobial resistance, virulence, metal resistance, and pangenome composition amongst isolates, clustering based on the presence and absence matrices for each were assessed by grouping factors of treatment group, diarrhea status, sequence type, and antibiotic exposure as previously described (Lee et al., 2023). A PERMDISP2 procedure was conducted to evaluate if dispersions of groups for each grouping factor were homogenous (Anderson, 2006; Anderson et al., 2006). Permutational analysis of variance (PERMANOVA) and ANOSIM (analysis of similarity) were then performed to evaluate equivalence of centroids of groups and average of ranks of within-group to between-group distances, respectively (Anderson and Walsh, 2013). Additionally, non-metric multidimensional scaling was performed by grouping factor of sequence type for AMR and virulence genes. All tests were performed using 10,000 permutations and a Jaccard distance metric in R using the vegan package (Oksanen et al., 2022).

Logistic regression models were constructed to assess the association between the presence of ARGs with calf-level factors. Models were constructed with outcomes specified as the presence or absence of individual ARGs, quinolone resistance determinants (presence of any point mutations or plasmid-mediated quinolone resistance determinants), and extended spectrum beta-lactamase (ESBL) resistance genes. ARGs which were present in all *E. coli* genomes were omitted from analysis. Calf-level factors included in model building included treatment group (isolate from zinc – or placebo – treated calf), therapeutic spectinomycin exposure at the time of isolate collection, and diarrhea status of the calf at the time of isolate collection. Antibiotic exposure and calf diarrhea status were evaluated based on individual calf-level data collected through daily assessments. Specifically, spectinomycin treatment was coded as a binary variable (received treatment or not), number of doses received (0, 1, or 2 doses), or days from the last spectinomycin dose received, and diarrhea status was coded as days on or from diarrhea or a categorical variable (healthy/pre-diarrheic, diarrheic, or recovered). Final models were selected based on the lowest AIC after inclusion of confounders (antimicrobial exposure for all ARG models) and any other significant predictors. Given their public health significance, the association between the presence of extended spectrum beta-lactamase (ESBL) genes and other ARGs were also evaluated using Fisher’s exact test.

Antimicrobial susceptibility testing data previously collected on study isolates (broth microdilution using the NARMS Gram Negative panel, YCMV3AGNF) were used to assess the concordance between genotypic and phenotypic resistance (Lee et al., 2022) for the following drugs: gentamicin, streptomycin, amoxicillin-clavulanic acid, cefoxitin, ceftiofur, ceftriaxone, trimethoprim-sulfamethoxazole, azithromycin, ampicillin, chloramphenicol, nalidixic acid, ciprofloxacin, and tetracycline. Classification of isolates into susceptible, intermediate, and resistant categories were conducted using CLSI breakpoints, with the exception of streptomycin and azithromycin where NARMS breakpoints were used due to lack of CLSI breakpoints (Supplementary file). Additionally, sulfisoxazole was omitted from analysis as resistance could not be determined due to the limited range of drug dilutions in the panel. Multidrug-resistance (MDR) was defined as resistance to ≥1 drug in ≥3 antimicrobial classes (Magiorakos et al., 2012). Concordance included phenotypically resistant isolates with the corresponding ARG(s) (TP, true positive) and phenotypically susceptible isolates with absence of corresponding ARG(s) (TN, true negative). Discordance included phenotypically resistant isolates not having the corresponding ARG(s) (FN, false negative), and phenotypically susceptible isolates having the corresponding ARG(s) (FP, false positive). Sensitivity and specificity were evaluated as TP/(TP + FN) and TN/(TN + FP), respectively. For analysis, intermediate isolates were grouped with susceptible isolates.

To evaluate the co-occurrence of plasmid replicons and ARGs, a pairwise co-occurrence matrix was constructed and visualized as networks using Gephi (Bastian et al., 2009) as previously described (Lee et al., 2023). To assess the linkage patterns of ARGs and metal resistance genes, pairwise probabilistic co-occurrence analysis was conducted using default settings in the R package co-occur (Griffith et al., 2016).

## 3 Results

### 3.1 WGS of MDR *Escherichia coli* isolates

*Escherichia coli* genomes in this study had an average of 193 contigs, coverage of 112X, and quality score of 38. Additional quality metrics, AST data, and metadata of genomes in this study are available in the Supplementary file.

### 3.2 Concordance of AMR phenotypes with genotypes

*Escherichia coli* isolates in this study were previously determined to be MDR through AST. To assess AMR concordance, predictions of AMR phenotype from genotype was evaluated for 13 drugs using previously collected AST data. Across all tested drugs, genotypic AMR predicted phenotypic AMR with an overall sensitivity of 100% and specificity of 98.58% (Table 1). Discordances in specificity included a streptomycin susceptible isolate with a streptomycin resistance gene (*aadA2*), and a ceftiofur intermediate isolate with carriage of an AmpC beta-lactamase gene (*bla*_CMY2_).

**Table 1:** Genotypic prediction of phenotypic resistance in dairy calf *E. coli* isolates (*n* = 43).

CLSI class	Antimicrobial agent	Phenotype: susceptible		Phenotype: resistant		Sensitivity^b^ (%)	Specificity^c^ (%)
		(No. of isolates)		(No. of isolates)			
		Genotype: resistant	Genotype: susceptible	Genotype: resistant	Genotype: susceptible		
		(FP)^a^	(TN)^a^	(TP)^a^	(FN)^a^		
Aminoglycosides	GEN	0	5	38	0	100%	100%
	STR	1	0	42	0	100%	0%
B-lactam combination agents	AUG2	0	2	41	0	100%	100%
Cephems	FOX	0	2	41	0	100%	100%
	XNL	1	1	41	0	100%	50%
	AXO	0	1	42	0	100%	100%
Folate pathway antagonists	SXT	N/A	N/A	43	0	100%	0%
Macrolides	AZI	0	40	N/A	N/A	N/A	100%
Penicillins	AMP	N/A	N/A	43	0	100%	N/A
Phenicols	CHL	0	10	33	0	100%	100%
Quinolones	NAL	0	39	4	0	100%	100%
	CIP	0	39	4	0	100%	100%
Tetracyclines	TET	N/A	N/A	43	0	100%	N/A
Overall	–	2	139	415	0	100%	98.58%

a FP false positive; TN true negative, TP true positive, FN false negative.

b Sensitivity TP/(TP + FN).

c Specificity TN/(TN + FP).

### 3.3 *Escherichia coli* genome population structure

**Figure 1:**
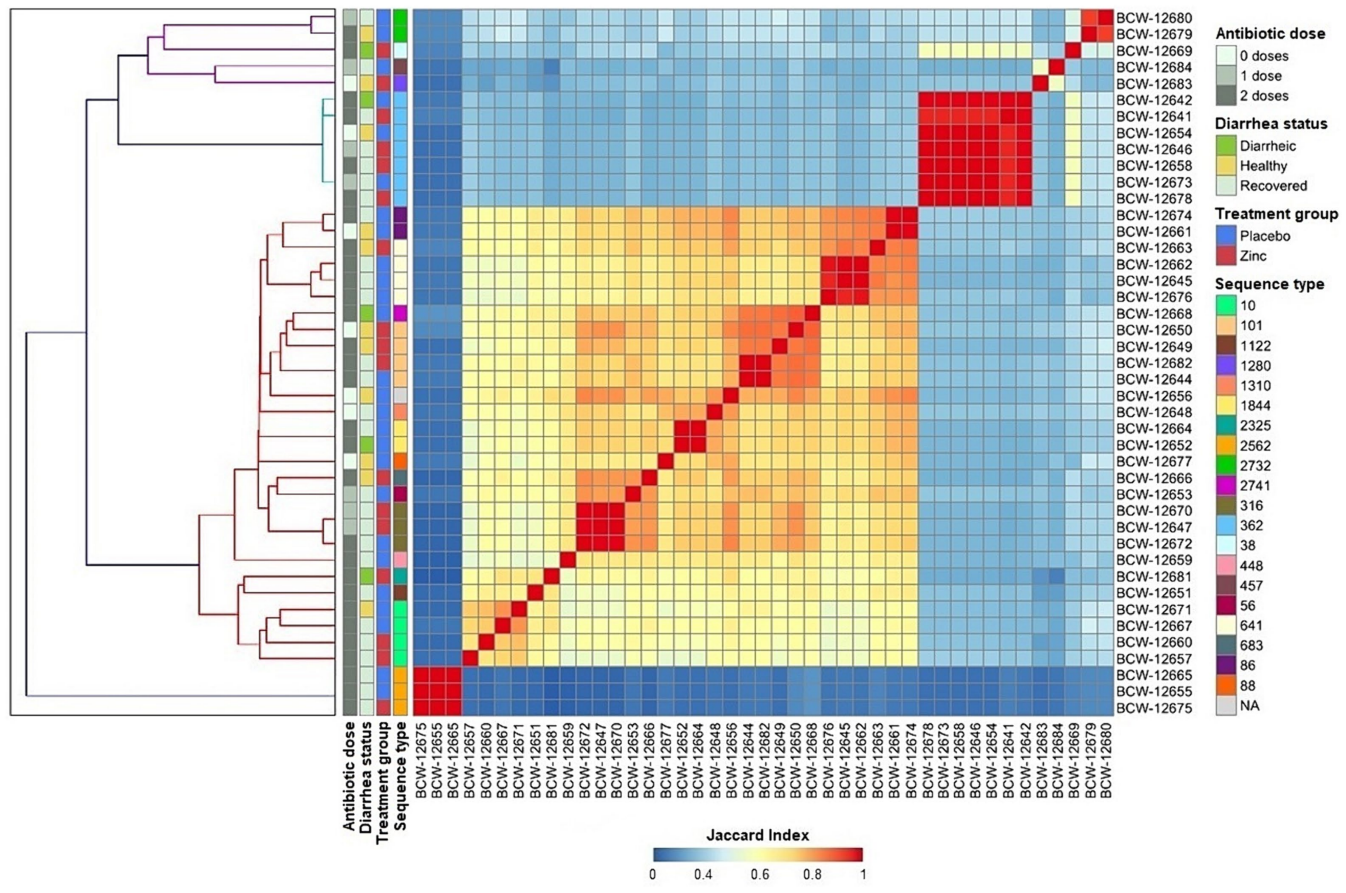
All-by-all comparison of genome similarity of *E. coli* isolates (*n* = 43) from pre-weaned dairy calves, generated using MinHash sketches from draft whole-genome assemblies of k-mers with a length of 31 and sketch size of 100,000. The heatmap color gradient corresponds to the Jaccard Similarity Index (JSI) for each pairwise comparison, with values close to 0 and 1 corresponding to high genome dissimilarity and similarity, respectively.

**Figure 2:**
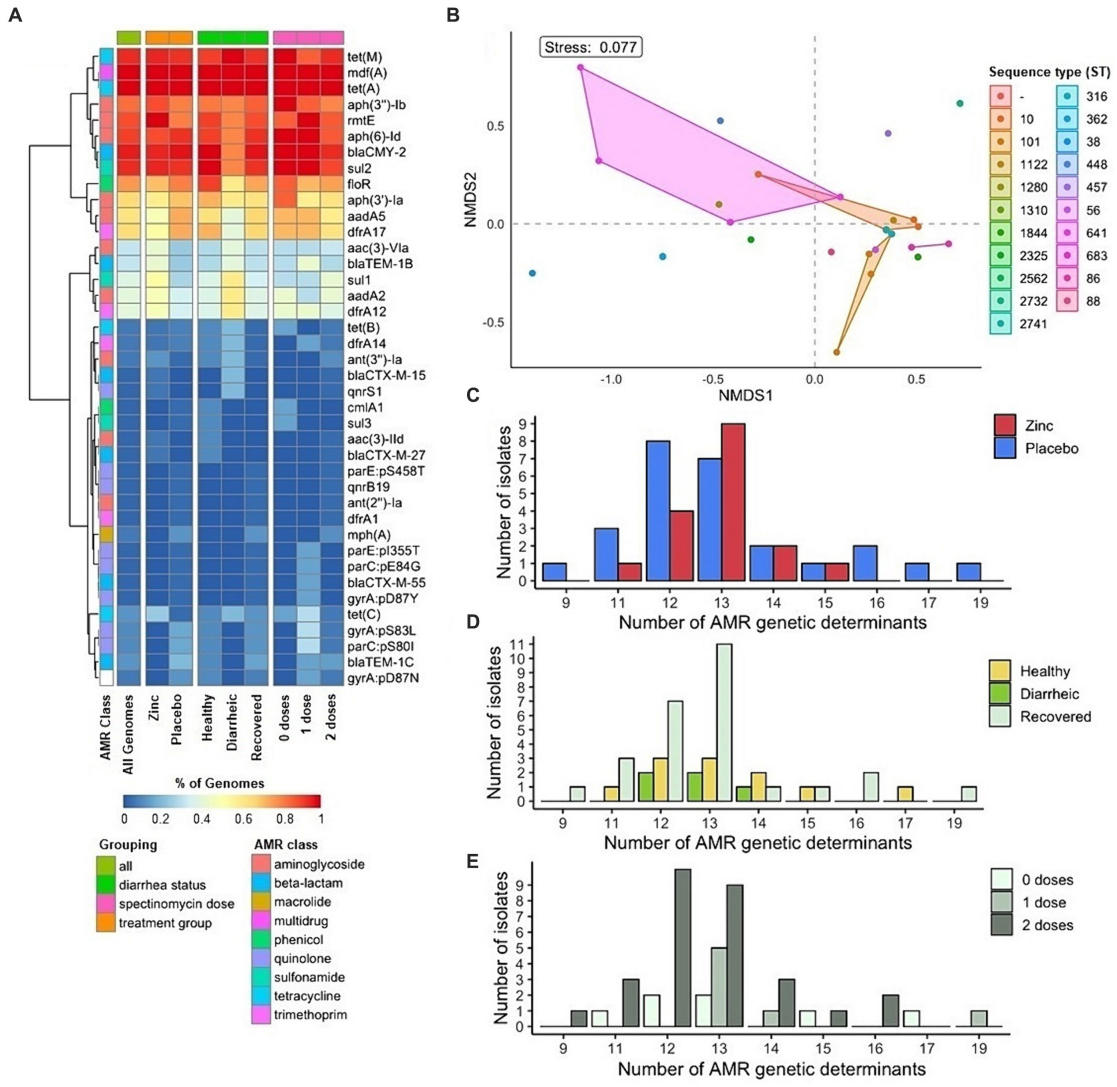
Antimicrobial resistance genetic determinants in fecal *E. coli* isolates from pre-weaned dairy calves (*n* = 43). **(A)** Heat map of ARG prevalence among isolates. **(B)** Non-metric multidimensional scaling of ARG composition of isolates by grouping factor of sequence type. Distribution of number of ARGs in *E. coli* isolates by **(C)** treatment group **(D)** diarrhea status and **(E)** therapeutic antibiotic exposure.

**Figure 3:**
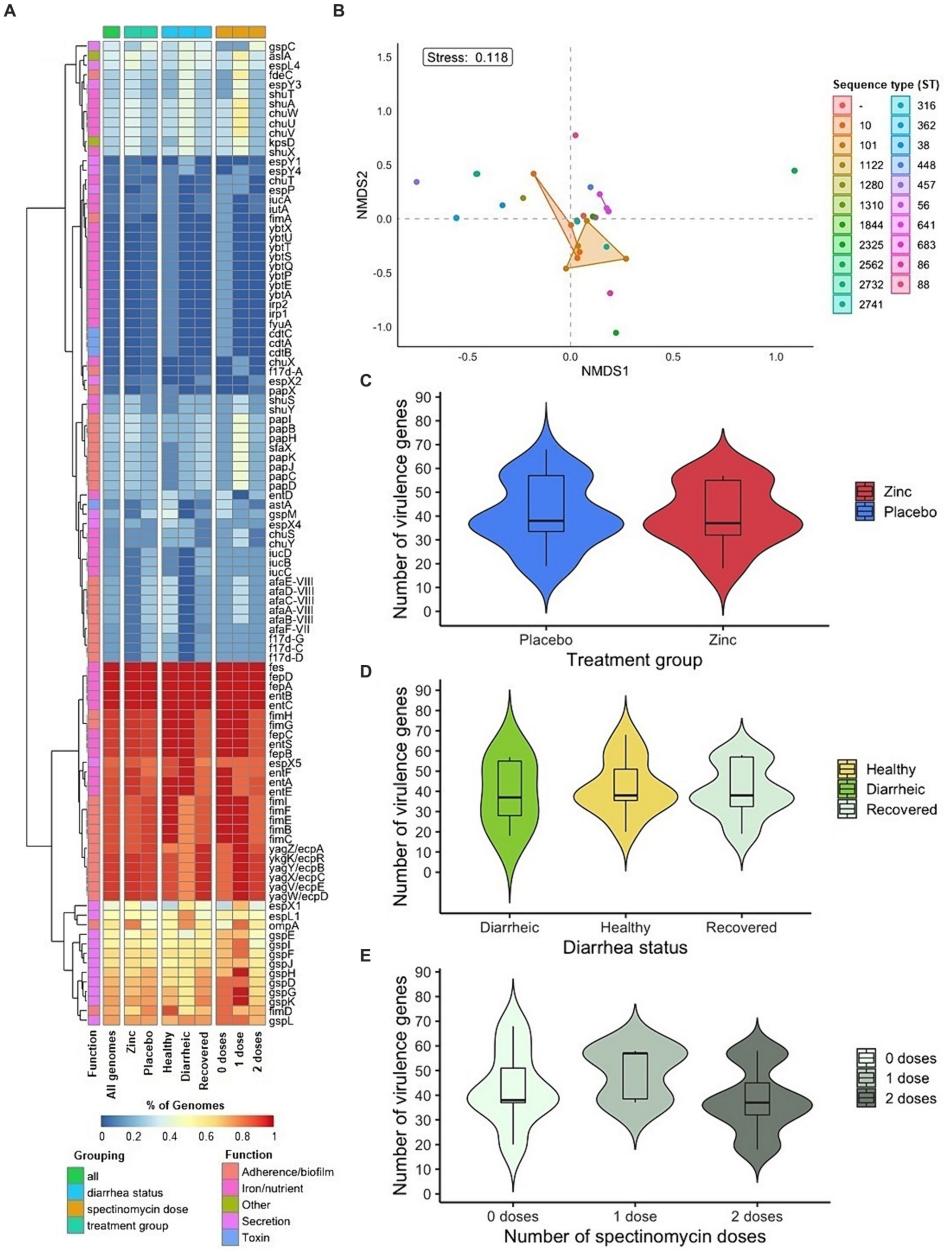
Virulence genes in fecal *E. coli* isolates from pre-weaned dairy calves (*n* = 43). **(A)** Heat map of virulence gene prevalence among isolates. **(B)** Non-metric multidimensional scaling of virulence gene composition in isolates by grouping factor of sequence type. Distribution of number of virulence genes in *E. coli* isolates by **(C)** treatment group **(D)** diarrhea status and **(E)** therapeutic antibiotic exposure.

Whole genome analysis of the isolates revealed a large genomic diversity of *E. coli* genomes. All-by-all comparison identified three main clusters that exhibited minimal to no relationship to calf disease status (healthy, diarrheic, or recovered calves), treatment group (placebo or zinc), or therapeutic antimicrobial treatment (0, 1, or 2 doses of spectinomycin). A total of 20 unique sequence types (STs) based on the 7-gene allelic profile were identified among 42 isolates, with one isolate unable to be assigned to a ST. The most frequently occurring STs included ST362 (7/43, 16.28%), followed by ST10 (4/43, 9.30%), ST101 (4/43, 9.30%), and ST641 (4/43, 9.30%). STs correlated with group and individual clusters from whole genome comparisons, with distinctive variability in genome content observed within each ST (Figure 1), indicating that the genes used to define ST were stable, but the remainder of the genome contained large variations. Specifically, the most prevalent STs exhibited substantial heterogeneity in genome composition, particularly ST10, ST101, and ST641 which had variable accessory genes including those for AMR and virulence (Figures 1, 2B, 3B). This observation indicated that WGS provided higher resolution characterization of strain variation than MLST, and prompted examining the pangenome for better understanding of the gene variation among isolates in this study.

### 3.4 Pangenome analysis of *Escherichia coli* isolates

The pangenome of *E. coli* isolates in this study was open and comprised of 14,011 genes that included a core genome with 3,117 genes and a soft-core, shell, and cloud genomes of 219, 3,076, and 7,599 genes, respectively. Analysis of the cumulative gene curve representing the number of total homologous genes and conserved homologs indicated an open pangenome that was covered with approximately 10 genomes within this population (Supplementary Figure S1). While the core was represented within a smaller portion of the isolates, genes from the variable portion of the pangenome represented 77.75% variation in the isolate population.

### 3.5 AMR, virulence, metal resistance, and pangenome profiles and diversity

The collective ARGs, virulence genes, metal resistance genes, and pangenome elements of *E. coli* were evaluated using multivariate analysis to assess if variability in these genomic profiles were driven by host-level factors. Tests for differences in *E. coli* genomic content for AMR, virulence, metal resistance, and pangenome elements indicated that dispersion differences were not significantly different among isolates by treatment group, diarrhea status, and therapeutic antibiotic exposure (PERMDISP2 *p* > 0.05, Table 2). Additionally, grouping factors evaluated in this study accounted for a low proportion of variance in AMR, virulence, metal resistance, and pangenome composition in *E. coli* genomes (PERMANOVA R^2^ = 9.39E-3-0.04), with equal or greater dissimilarities in average of ranks within group than those of between-groups across all factors (ANOSIM R = ~0 or R < 0) (Table 2). These analyses indicated that the host-level factors evaluated in this study—diarrheal disease status, dietary zinc supplementation, and antibiotic treatment—had minimal influence on the genomic composition of *E. coli*. These findings provided impetus to evaluate the distribution of genes individually with respect to host-level factors.

**Table 2:** Results of PERMDISP2, PERMANOVA, and ANOSIM tests.

Group	PERMDISP2 *p*-value (F)	PERMANOVA *p*-value (R^2^)	ANOSIM *p*-value (R)
AMR (ResFinder) genes (*n* = 40)			
Treatment group	0.75 (0.10)	0.15 (0.04)	0.38 (4.23E−3)
Diarrhea status	0.74 (0.29)	0.87 (0.02)	0.49 (−5.89E−3)
Antibiotic doses	0.62 (0.48)	0.83 (9.39E−3)	0.85 (−0.09)
Virulence (VFDB) genes (*n* = 103)			
Treatment group	0.86 (0.03)	0.73 (0.01)	0.75 (−0.035)
Diarrhea status	0.98 (0.02)	0.76 (0.03)	0.57 (−0.020)
Antibiotic doses	0.69 (0.38)	0.70 (0.02)	0.67 (−0.048)
BacMet genes (*n* = 153)			
Treatment group	0.71 (0.15)	0.22 (0.03)	0.37 (7.93E−3)
Diarrhea status	0.96 (0.04)	0.93 (0.02)	0.49 (−4.04E−3)
Antibiotic doses	0.97 (0.03)	0.76 (0.01)	0.52 (−0.01)
Pangenome (Roary) elements (*n* = 14,011)			
Treatment group	0.92 (0.01)	0.34 (0.025)	0.42 (2.0E–3)
Diarrhea status	0.59 (0.54)	0.93 (0.03)	0.80 (−0.066)
Antibiotic doses	0.71 (0.35)	0.57 (0.02)	0.47 (2.63E–3)

### 3.6 Antimicrobial resistance genetic determinants (ARGs)

Across the 43 *E. coli* genomes, a total of 40 ARGs among diverse antimicrobial classes were detected. The average and median number of ARGs per genome—including SNPs for quinolone resistance—was 13 ARGs with a range of 9 to 19. ARGs conferring resistance to antimicrobials of public health significance included seven SNPs in chromosomal genes—pS83L, pD87N, and pD87Y in *gyrA*, pS80I and pE84G in *parC*, and pI355T and pS458T in *parE*—and 2 plasmid-mediated quinolone resistance genes—*qnrB19* and *qnrS1*—associated with quinolone resistance, and those for AmpC (*bla*_CMY-2_) and extended-spectrum (*bla*_CTX-M-15_, *bla*_CTX-M-27_, and *bla*_CTX-M-55_) beta-lactamases (ESBL). The presence of ESBL gene(s) in *E. coli* was significantly associated with the presence of one or more quinolone resistance determinants (*p* < 0.05, Fisher’s exact test).

ARGs present in more than half of the isolates included *mdf*(A) (43/43, 100%), *aph(6)-Id* (39/43, 90.7%), *rmtE* (38/43, 88.4%), *aph(3″)-Ib* (36/43, 83.7%), and *aadA5* (27/43, 62.8%) for aminoglycoside resistance, *bla*_CMY-2_ (41/43, 95.3%) for beta-lactam resistance, *dfrA17* (28/43, 65.1%) for trimethoprim resistance, *floR* (33/43, 76.7%) for phenicol resistance, *sul2* (40/43, 93.0%) for sulfonamide resistance, and *tet*(A) (43/43, 100%) and *tet*(M) (40/43, 93.0%) for tetracycline resistance (Figure 2A). The average number of ARGs across all genomes and collective AMR profiles did not differ significantly by dietary zinc supplementation treatment group (zinc or placebo), diarrhea status (healthy, diarrheic, and recovered), and therapeutic antibiotic exposure (0, 1, or 2 doses) (Table 2, Figures 2C–E).

### 3.7 Mobile genetic elements associated with ARGs

**Figure 4:**
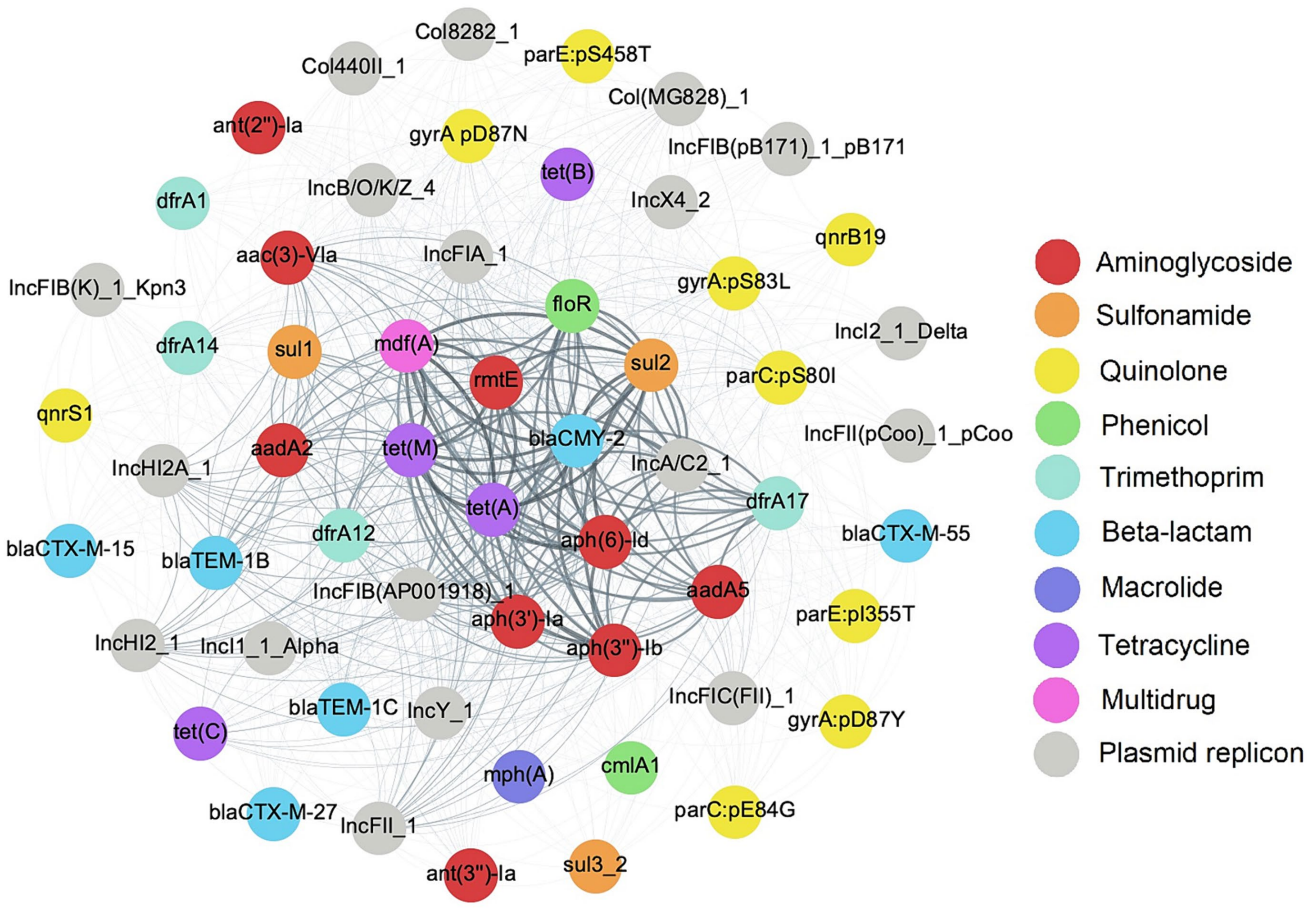
Co-occurrence network of plasmid replicons and antimicrobial resistance genetic determinants (ARGs) in *E. coli* isolates. Nodes representing ARGs are color coded by antimicrobial class and edges representing low to high frequency of co-occurrence are depicted in a light to dark color gradient.

As *E. coli* isolates in this study were MDR, it was of interest to investigate the mobile genetic elements associated with ARGs that may contribute to AMR co-transfer. Eighteen putative plasmids based on the presence of plasmid replicons were identified across all genomes, with a pairwise co-occurrence matrix indicating high frequency co-occurrence of AMR gene clusters with certain putative plasmids (Figure 4). The most frequently co-occurring gene network of *aph(6)-Id*, *bla*_CMY-2_, *floR*, *mdf*(A), *sul2*, and *tet*(A) was associated with the IncA/C2 plasmid replicon in 30 (69.8%) genomes. A second smaller network including *mdf*(A), *rmtE*, and *tet*(A) co-occurred with the IncFIB (AP001918) plasmid replicon at a frequency of 20 (46.5%) genomes. At a minimum threshold co-occurrence of ≥10 genomes (about 25% of the genomes), a larger network of genes including *aac(3)-VIa*, *aadA2*, *dfrA12*, *mdf*(A), *rmtE*, *sul1*, *tet*(A), and *tet*(M) were detected with IncHI2/2A plasmid replicons. Screening for plasmid replicons among genomes in this study identified unique sets of ARGs in co-occurrence with primarily large AMR plasmids.

### 3.8 Association between dietary zinc supplementation and genotypic AMR

**Figure 5:**
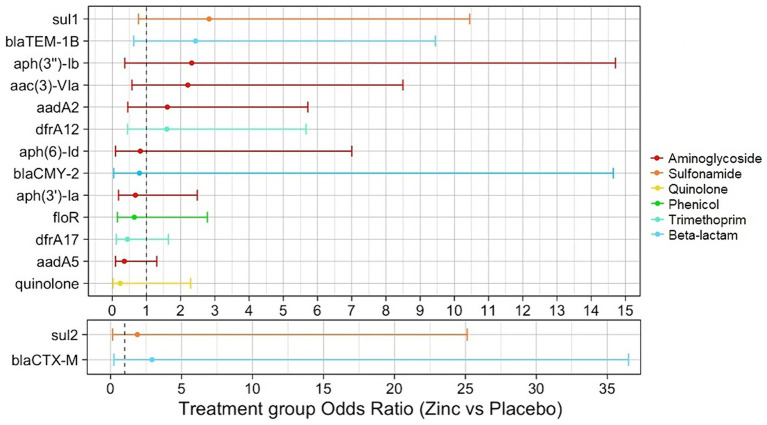
Antibiotic exposure-adjusted logistic regression models evaluating the association between presence of antimicrobial resistance genetic determinants (ARGs) and calf treatment group of *E. coli* isolates. Point estimates for each model are color-coded by antimicrobial class. Binary outcomes for quinolone and *bla*_CTX-M_ models were specified as the presence/absence of any quinolone resistance mechanism (plasmid-mediated genes or point mutations) and the presence/absence of any *bla*_CTX-M_ alleles, respectively.

The relationship between genotypic AMR and calf zinc treatment group of isolates was examined to determine the association between dietary zinc supplementation in pre-weaned dairy calves and the selection of specific ARGs. From descriptive analysis, SNPs in genes for quinolone resistance were exclusively detected in isolates from placebo calves. Antibiotic exposure-adjusted logistic regression models identified higher odds of certain ARGs in *E. coli* isolates from zinc-treated compared to placebo calves (*dfrA12*, *aadA2*, *sul2*, *aac(3)-VIa*, *aph(3″)-Ib*, *bla*_TEM-1B_, *sul1*, and alleles of *bla*_CTX-M_), though none of these associations were significant (OR = 1.60–2.92, *p* > 0.05). Conversely, there were non-significant lower odds for other ARGs and point mutations associated with quinolone resistance for isolates from zinc-treated to placebo calves [*aadA5*, *dfrA17*, *floR*, *aph(3′)-Ia*, *bla*_CMY-2_, *aph(6)-Id* (OR = 0.23–0.82, *p* > 0.05)] (Figure 5, Table 3, Supplementary Tables S1–S15).

**Table 3:** Distribution of antimicrobial resistance genetic determinants (ARGs) in *E. coli* by calf treatment group and treatment group point estimates (zinc vs. placebo) from antibiotic exposure-adjusted logistic regression models.

ARG	Zinc	Placebo	OR (95% CI)	p-value
*sul1*	9/17 (52.94%)	7/26 (26.92%)	2.83 (0.77, 10.45)	0.12
*bla*_TEM-1B_	7/17 (41.18%)	6/26 (23.08%)	2.44 (0.63, 9.45)	0.2
*aph(3″)-Ib*	15/17 (88.24%)	21/26 (80.77%)	2.32 (0.37, 14.71)	0.37
*aac(3)-VIa*	7/17 (41.18%)	6/26 (23.08%)	2.21 (0.58, 8.50)	0.25
*aadA2*	8/17 (47.06%)	9/26 (34.62%)	1.61 (0.45, 5.72)	0.46
*dfrA12*	8/17 (47.06%)	9/26 (34.62%)	1.60 (0.45, 5.67)	0.47
*aph(6)-Id*	15/17 (88.24%)	24/26 (92.31%)	0.82 (0.096, 7.00)	0.86
*floR*	12/17 (70.59%)	21/26 (80.77%)	0.65 (0.15, 2.78)	0.56
*dfrA17*	9/17 (52.94%)	19/26 (73.08%)	0.45 (0.12, 1.64)	0.22
*aadA5*	8/17 (47.06%)	19/26 (73.08%)	0.35 (0.096, 1.30)	0.12
quinolone	1/17 (5.88%)	5/26 (19.23%)	0.23 (0.024, 2.29)	0.21
*sul2*	16/17 (94.12%)	24/26 (92.31)	1.89 (0.14, 25.12)	0.63
*bla*_CTX-M_	2/17 (11.76%)	1/26 (3.85%)	2.92 (0.23, 36.49)	0.41

Binary outcomes for quinolone and *bla*_CTX-M_ models were specified as the presence/absence of any quinolone resistance mechanism (plasmid-mediated genes or point mutations) and the presence/absence of any *bla*_CTX-M_ alleles, respectively.

### 3.9 Virulence genes

A total of 103 virulence genes corresponding to adherence/biofilm formation (*n* = 36), iron/nutrient acquisition (*n* = 40), secretion (*n* = 21), toxin (*n* = 4), and other functions (*n* = 2) were detected across *E. coli* genomes. The average and median number of virulence genes were 40.58 and 38, respectively (range of 18 to 68). Five virulence genes related to enterobactin (*entB*, *entC*, *fepA*, *fepD* and *fes*) were detected across all isolates (Figure 3A). The number of virulence genes and collective virulence profiles across genomes did not differ significantly by dietary zinc supplementation treatment group, diarrhea status, and therapeutic antibiotic exposure (Table 2, Figures 3C–E).

Virulence genes from the *afa-7* and *afa-8* clusters (*afaA-E*) encoding afimbrial adhesins were detected primarily in isolates from placebo calves (85.71%, 6/7), with the full gene set present in six isolates. Other virulence genes detected related to colonization included those encoding F17 fimbriae (*f17d-A*, *f17d-C*, *f17d-D*, and *f17d-G*) in six isolates, and P fimbriae in 12 isolates (*pap* genes) (Bertin et al., 2000; Bihannic et al., 2014; Ryu et al., 2020). Additionally, genes in the *fim* cluster (*fimA-I*) encoding type 1 fimbriae were present in the majority of isolates, though only one isolate harbored the *fimA* structural gene and three isolates the *fimH* adhesin gene. Major virulence genes related to secretion included those corresponding to Type II (*gsp*) and Type III (*esp*) secretion systems. Virulence genes for toxins, *astA* (enteroaggregative heat-stable enterotoxin) and/or *cdtABC* (cytolethal distending toxin), were identified in isolate(s) from pre–and post-diarrheic calves. Overall, virulence genes were interspersed in the population across calf zinc treatment group and diarrhea status. The largest number and diversity of virulence genes identified corresponded to iron/nutrient acquisition, including genes *chuSTUVWXY* (heme uptake), *entA-F* (enterobactin), *fepA-D* (enterobactin), *fyuA* (yersiniabactin receptor), *iucABCD-iutA* (aerobactin), and those in the *ybt* operon (yersiniabactin) (Figure 3A).

### 3.10 Association between AMR and metal resistance genes

**Figure 6:**
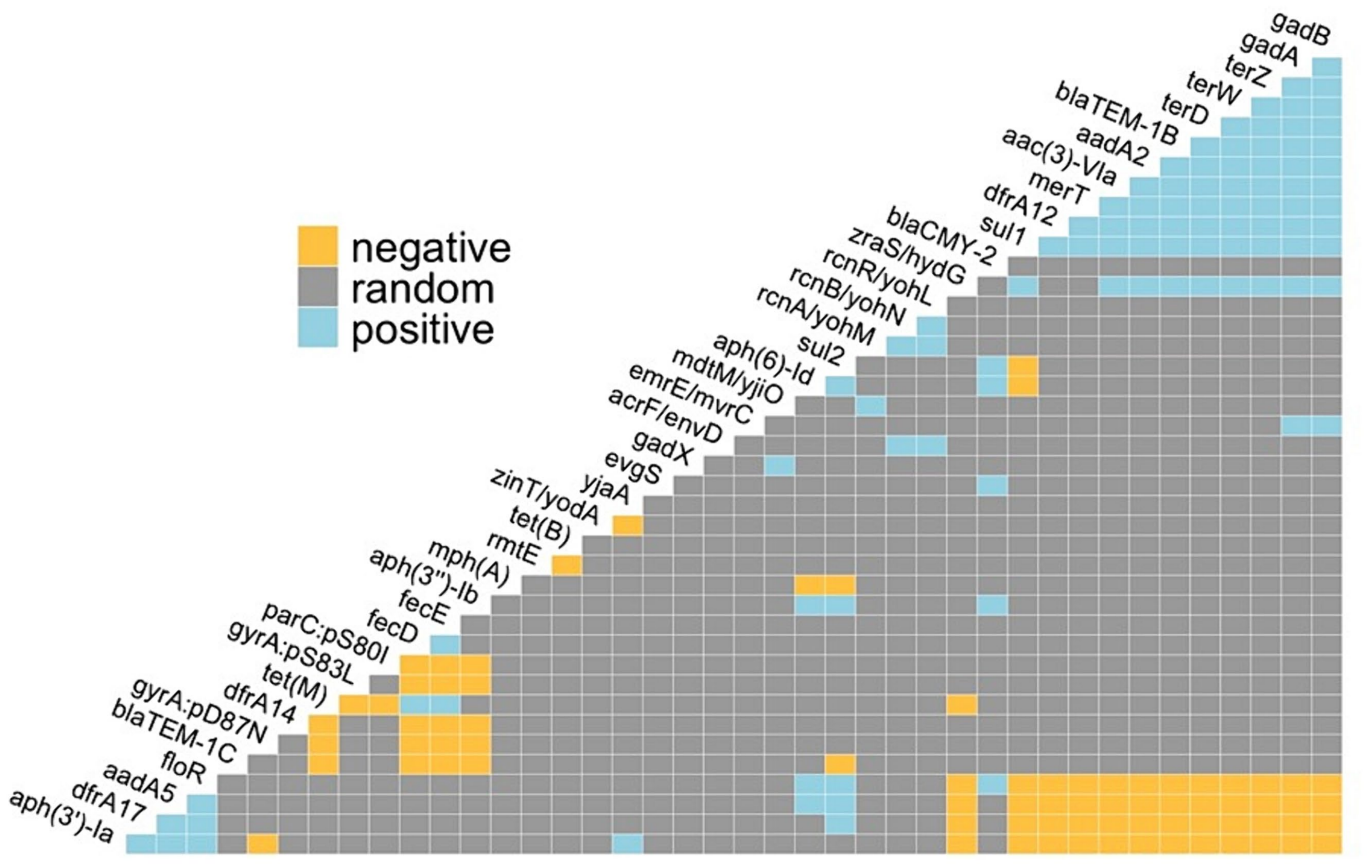
Pairwise probabilistic co-occurrence analysis for positive, negative, or random associations between antimicrobial resistance genetic determinants and metal resistance genes in *E. coli* isolates.

A total of 153 metal resistance genes (MRGs) were identified across all *E. coli* genomes examined, with the average and median number of MRGs per genome being 128.42 and 127, respectively, with a range of 123 to 135. Co-occurrence analysis of ARGs and metal resistance genes included 16,585 gene pairs and identified 96 positive and 77 negative co-occurrences. Positive associations including both ARGs and metal resistance were observed between aminoglycoside (*aac(3)-VIa*, *aadA2*), beta-lactam (*bla*_TEM-1B_), sulfonamide (*sul1*), and trimethoprim (*dfrA12*) resistance and acid (*gadA* and *gadB*), tellurium (*terZ* and *terW*) and mercury (*merT*) resistance genes (Figure 6).

## 4 Discussion

The enteric microbiota serves as a symbiotic partner of the host, with crucial roles in intestinal health, metabolism, and host immune response (Casadevall and Pirofski, 2000; Kaiko and Stappenbeck, 2014; Jandhyala et al., 2015). The acquisition and loss of genes—such as those for AMR—in enteric microbes like *E. coli* can occur as adaptive responses to environmental (e.g., dysbiosis) and host changes (e.g., diet and disease). In this study, we investigated the host-microbe interface of enteric MDR *E. coli* from pre-weaned dairy calves to evaluate potential contributing factors to MDR persistence and better understand the relationship between genomic composition and host-level factors of antimicrobial exposure, dietary zinc supplementation, and calf diarrheal disease.

Whole genome sequence analysis revealed high genome variability and an open pangenome of multidrug-resistant (MDR) *E. coli* from dairy calves in this study. The diverse population structure of *E. coli* has been well-documented, with the frequent acquisition, loss, and modification of genes contributing to its large gene pool, fitness, and competitive ability to thrive in widespread geographical and host environments (Horesh et al., 2021). From all-by-all comparisons of the WGS, the isolates in this study clustered by sequence type (ST) but not host-level factors of disease status, dietary influences, or antimicrobial exposure. Common STs identified included ST362, a frequently occurring ST in calves that has been associated with extra intestinal infections (Falgenhauer et al., 2017; Vieille et al., 2019; Homeier-Bachmann et al., 2022). Other prevalent STs were those with zoonotic potential, such as ST641 which has been isolated from poultry and goat sources (Cortés et al., 2010; Zhuge et al., 2021; Treilles et al., 2023), ST10, a widespread lineage of pathogenic and commensal *E. coli* which are prominently MDR in animal populations (Haley et al., 2023; Silva et al., 2023; Wang et al., 2023), and ST101, another frequently occurring MDR clonal group frequently detected in food, water, food animal, and human matrices (Umpiérrez et al., 2017; Zhong et al., 2019; Sauget et al., 2023; Silva et al., 2023).

The accessory genome of *E. coli* encodes various characteristics for survival and reproduction, including those related to AMR (Hall et al., 2021). The early developing microbiota of calves has been observed to harbor high prevalence and diversity of ARGs (Liu et al., 2019; Haley et al., 2023), which is corroborated by the large assortment of ARGs detected in our *E. coli* genomes. In this study, the number of ARGs in *E. coli* did not correlate with antimicrobial use. However, the presence of ARGs corresponding to certain antimicrobial classes were consistent with the AMR selection pressures in our study; the high prevalence and diversity of tetracycline and aminoglycoside ARGs detected in *E. coli* genomes was reflective of the tetracycline and neomycin administered in dietary milk and spectinomycin for the therapeutic treatment of diarrhea. The same tetracycline and aminoglycoside ARGs in our study are frequently present in *E. coli* from calves (Jeamsripong et al., 2021; Salaheen et al., 2023), even from those with no previous antibiotic exposure (Liu et al., 2019). Similarly, other ARGs identified in our study including those encoding beta-lactam, phenicol, trimethoprim, and/or sulfonamide resistance have also been previously reported in calves (Haley et al., 2023; Salaheen et al., 2023), suggesting that the collection of ARGs in our study is representative of the enteric calf resistome during early life.

A major mechanism of third-generation cephalosporin resistance in *Salmonella* and *E. coli* from food and food-producing animals is AmpC-type beta-lactamase *bla*_CMY-2_, which was detected in almost every *E. coli* genome in this study, despite the lack of beta-lactam use in calves. The occurrence of *bla*_CMY-2_ in dairy cattle has been presumed to be from frequent use of ceftiofur for the intramammary treatment of mastitis and parenteral treatment of acute metritis and bacterial pneumonia (Durel et al., 2019). However, studies have found limited evidence for the direct dissemination of *bla*_CMY-2_ through ceftiofur use (Daniels et al., 2009; Schmidt et al., 2013) or associations between recent ceftiofur treatment and reduced-susceptible *E. coli* at the individual cow level (Tragesser et al., 2006). We found a high frequency of a co-occurrence networks with *bla*_CMY-2_, ARGs corresponding to aminoglycoside, phenicol, sulfonamide, and tetracycline resistance, and the IncA/C2 plasmid replicon in our study isolates. These data support observations from other studies, in which the occurrence of *bla*_CMY-2_ in absence of cephalosporin use has been postulated to be from its acquisition on large MDR plasmids, followed by clonal expansion and/or the presence of indirect and co-selective AMR pressures that maintain these plasmids at the herd-level (Alcaine et al., 2005; Subbiah et al., 2011; Martin et al., 2012; Schmidt et al., 2013; Deng et al., 2015). Other beta-lactam ARGs conferring resistance to cephalosporins found in this study included ESBL genes from the *bla*_CTX-M_ family (*bla*_CTX-M-15_, *bla*_CTX-M-27_, and *bla*_CTM-M-55_) from three *E. coli* genomes. In addition to being resistant to third-generation cephalosporins, ESBL – producing *E. coli* have important clinical consequences as they are frequently MDR to other critically important antimicrobials such as quinolones (Zurfluh et al., 2014; Azargun et al., 2018; Furmanek-Blaszk et al., 2023), a finding that is corroborated through the significant association observed between the presence of ESBL and quinolone resistance determinants among *E. coli* in this study.

While antimicrobial use is perceived as a main driver of AMR, non-antimicrobial factors such as heavy metal exposure have also been recognized to influence AMR selection. Heavy metals such as zinc are frequently used as growth promoters or therapeutic agents in livestock species (Yazdankhah et al., 2014); for example, dietary zinc supplementation in pre-weaned calves may be used to reduce the burden of diarrheal disease and promote calf growth (Glover et al., 2013; Feldmann et al., 2019; Chang et al., 2020; Liu et al., 2023; Yu et al., 2023). Little is known on the influence of dietary zinc on genomic AMR in cattle, however a previous study in swine found that high zinc in feed (2.5 g/kg) significantly increased intestinal abundance of tetracycline and sulfonamide ARGs (Vahjen et al., 2015). As all *E. coli* genomes in our study had *tet* genes, we were unable to evaluate the selection of tetracycline ARGs. Adjusted logistic regression models found higher odds ratios for the presence of sulfonamide genes— *sul1* (OR = 2.83, 95% CI 0.77–10.45) and *sul2* (OR = 1.89, 95% CI 0.14–25.12)—in *E. coli* from zinc compared to those from placebo calves. Although these findings were not statistically significant, the direction of associations support the aforementioned findings of potential sulfonamide ARG selection from dietary zinc (Vahjen et al., 2015). We also found unique SNPs in the genes conferring quinolone resistance in isolates from placebo treated calves, suggesting an antagonistic effect of zinc on certain classes of ARGs. However, the lower odds ratio for the presence of quinolone ARGs from logistic regression in isolates from zinc compared to placebo treated calves (OR = 0.23 95% CI 0.02–2.29) was also not statistically significant. These non-significant findings may be attributed to the small sample size of isolates in our study that may have resulted in inadequate power to detect differences in addition to other uncharacterized variables. Hence, future studies employing larger sample sizes are needed to ascertain the relationship between zinc exposure and ARG selection, particularly for those in our study (*sul2*, *bla*_CMY-2_, *aph(3″)-Ib,* and *bla*_CTX-M_ alleles) with large confidence intervals for point estimates.

Beyond the selection of individual ARGs, co-selection of both ARGs and metal resistance genes may occur through co-resistance, a phenomenon where dissimilar mechanisms for both resistances are selected due to their genetic linkage (Wales and Davies, 2015). The linkage of ARGs and metal resistance genes has been well documented (Baker-Austin et al., 2006; Wales and Davies, 2015; Nguyen et al., 2019). Patterns in ARG and metal resistance gene co-occurrence have been observed to vary depending on the organism, host, and location (Poole, 2017). In our study, we identified several positive co-occurrences between ARGs encoding resistance to beta-lactam, sulfonamide, aminoglycoside and trimethoprim and metal resistance genes encoding mercury and tellurium resistance. Positive co-occurrences between these ARGs from the same classes of antimicrobials and mercury and tellurium resistance genes were previously reported in fecal *E. coli* from dairy herds from Pennsylvania (Haley et al., 2023). These data suggest that specific AMR and metal resistance genes are pervasive and selected for in dairy cattle and their farm environments irrespective of geographical location.

In addition to evaluating potential host-level drivers of AMR in calf *E. coli*, this study compared genotypic AMR—the presence of ARGs and point mutations conferring quinolone resistance—with phenotypic AMR data from antimicrobial susceptibility testing. Genotypic AMR exhibited a high degree of concordance with phenotypic AMR for genomically heterogeneous MDR isolates in this study. Despite the small sample size of isolates (*n* = 43) from one host (dairy calves) and source (single dairy farm), our findings are consistent with previous work evaluating genotypic and phenotypic concordance in *E. coli* and *Salmonella* from cattle and/or food animal sources (Tyson et al., 2015; McDermott et al., 2016; Carroll et al., 2021; Lee et al., 2022, 2023). Discrepancies for streptomycin and ceftiofur as observed in two isolates in this study have been frequently reported (Tyson et al., 2015; McDermott et al., 2016; Lee et al., 2023), and may be a result of lack of CLSI breakpoints for these veterinary drugs, technical variability in AST/WGS processes (e.g., 2-fold variations in MIC from AST at intermediate and resistant cut-off thresholds), and choice of classifying intermediate isolates. As an example of the latter, grouping of intermediate and susceptible isolates for analysis resulted in discrepancy of a ceftiofur immediate isolate in this study; the genotypic and phenotypic AMR for this isolate would have been congruent if intermediate isolates were instead treated as resistant.

Diarrheal disease status of calves was not significantly associated with genomic variability in this study, including virulence profiles. *E. coli* can be categorized into various pathotypes depending on the presence of certain virulence attributes (Kaper et al., 2004), with common pathotypes associated with neonatal calf diarrhea including enteropathogenic (EPEC), Shiga toxin-producing (STEC), enterotoxigenic (ETEC), and enteroaggregative (EAEC) *E. coli* (Awad et al., 2020). While MDR *E. coli* isolates in this study lacked the comprehensive virulence markers of these diarrheagenic pathotypes, they encoded a wide diversity of virulence genes that overlap with those in pathogenic strains. For instance, adhesin virulence genes observed in our study, *fim* and *pap* genes encoding Type I fimbriae and P fimbriae respectively, are associated with various pathotypes in both humans and animals (Bertin et al., 2000; Sarowska et al., 2019), and *f17* genes encoding F17 fimbriae and *afa-7* and *afa-8* gene clusters encoding afimbrial adhesion appear to be more host-specific and predominant in bovine *E. coli* associated with diarrhea and septicemia (Lalioui and Le Bouguénec, 2001; Bihannic et al., 2014; Shahrani et al., 2014). Additionally, detected in a few isolates were *cdtABC* and *astA* encoding cytolethal distending toxin (CDT) and enteroaggregative heat-stable enterotoxin (EAST1), which are typically present in EPEC and ETEC, respectively (Yamamoto and Echeverria, 1996; Osek, 2003; Gomes et al., 2016; Meza-Segura et al., 2017). The presence of some but not all pathotype-determining virulence genes in *E. coli* from our study suggests that the acquisition and loss of genes (e.g., from host and environmental influences) may dynamically contribute to the virulence potential of *E. coli* and their ability to cause diarrheal disease.

The most abundant virulence genes in MDR *E. coli* in this study were those involved in iron acquisition (e.g., sideophores and heme uptake). Iron plays a critical role in microbial metabolic processes and cell division, and its acquisition is an important host–microbe interaction that contributes to bacterial survival and pathogen infection (Caza and Kronstad, 2013; Nairz and Weiss, 2020). Previous studies identified several iron acquisition systems— some of which were also identified in our study (e.g., *iucABCD-iutA*)— to be significantly enriched in MDR bovine *E. coli* (Haley et al., 2023, 2024). Virulence factors and ARGs are essential for bacteria to overcome host immune responses and antimicrobial exposure, respectively. The simultaneous carriage of both in MDR *E. coli* may confer a fitness advantage in adverse conditions, promoting the co-selection and maintenance of these genes in MDR isolates as opposed to their susceptible counterparts (Beceiro et al., 2013). Moreover, the pre-weaned calf diet is primarily composed of milk, which is nutritionally negligible in iron and may contribute to a low-iron environment in the calf gut that has been hypothesized to favor the selection of MDR *E. coli* with more extensive repertoires of iron acquisition systems (Haley et al., 2023, 2024). During infection and disease, host-driven iron sequestration occurs as an immune defense strategy to inhibit the growth of pathogens (Beceiro et al., 2013; Nairz and Weiss, 2020). As *E. coli* genomes in our study were from pre-weaned calves in various stages of diarrheal disease (pre-diarrheic, diarrheic, and recovered), we hypothesize that host-mediated iron withdrawal is another factor which may further favor the survival of MDR *E. coli* with high iron-scavenging capacity.

In conclusion, our analysis indicated that the genomes of MDR *E. coli* from pre-weaned dairy calves were highly diverse and minimally driven by the host-level factors evaluated in this study (dietary zinc supplementation, therapeutic antimicrobial treatment, and diarrhea disease status). Key limitations include the relatively small sample size of isolates and the absence of a susceptible and/or non-MDR group of *E. coli* genomes for comparison. Future work that evaluates longitudinal effects would provide greater insight on the relationship between genomic diversity and factors such as disease—which may occur in progressive stages—and antimicrobial exposure, which can rapidly and transiently impact the gut microbiome. Our findings corroborate previous reports of MDR *E. coli* from calves harboring diverse ARGs conferring resistance to clinically important drugs, enriched abundance of virulence factors for iron acquisition systems, and linkage of certain metal resistance genes and ARGs. These data suggest that the selection and persistence of MDR *E. coli* in calves are adaptive and attributed to the presence of these and/or other unidentified genes that confer a fitness advantage in the calf gut.

## Data Availability

The datasets presented in this study can be found in online repositories. The names of the repository/repositories and accession number(s) can be found in the article/Supplementary material. WGS data for isolates in this study are available at the 100K Pathogen Genome Project BioProject (PRJNA1142533).
