# Supplementary material for: Whole genome sequence analysis reveals high genomic diversity and potential host-driven adaptations among multidrug-resistant Escherichia coli from pre-weaned dairy calves
Source: Front Microbiol. 2024 Sep 3;15:1420300. doi: 10.3389/fmicb.2024.1420300 (PMC11409426; doi:10.3389/fmicb.2024.1420300)
Supplement: Supplementary file 1 [file Data_Sheet_1.docx]

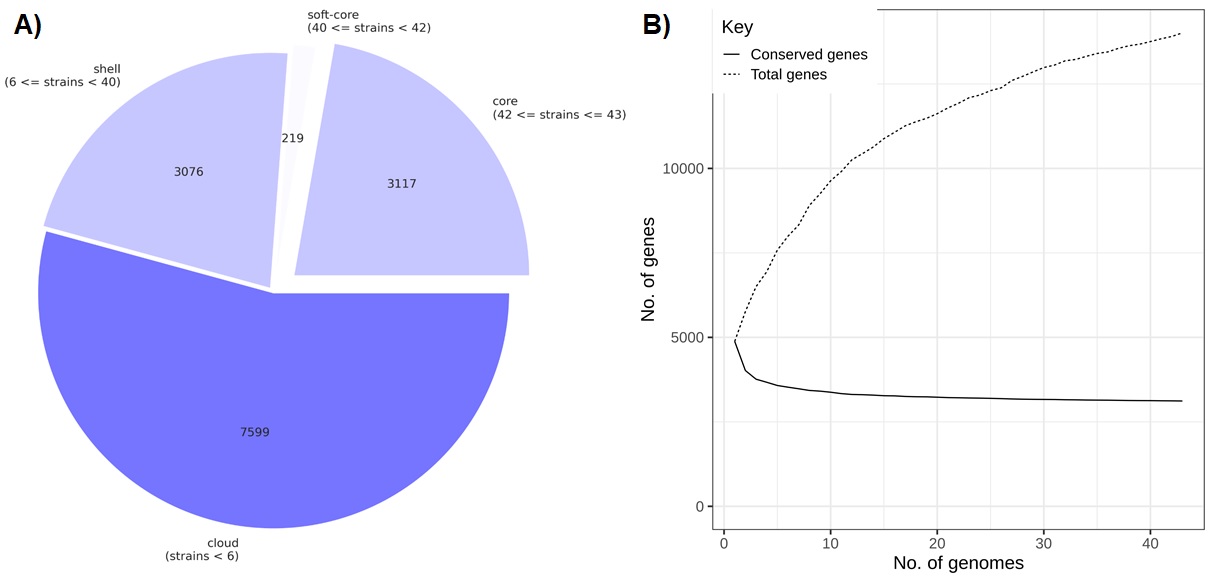


**Supplementary Figure 1**. Pangenome analysis of *E. coli* genomes (n=43), conducted using Roary. (a) Summary of *E. coli* pangenome (14,011 genes) (b) Gene accumulation curve of the number of conserved homologous genes (homologs) and the total distinct homologs of the *E. coli* pangenome, depicted by solid and dashed lines, respectively.

**Supplementary Table 1.** Final logistic regression model for the association between the presence of *bla*_CTX-M_ alleles in *E. coli* genomes and treatment group of calves.

| Factor | Level | Coefficient (SE) | Odds Ratio (95% CI) | P-value |
| --- | --- | --- | --- | --- |
| Treatment group | Zinc | 1.07 (1.29) | 2.92 (0.23, 36.49) | 0.41 |
|  | Placebo | Referent | - | - |
| Days from spectinomycin treatment | - | 0.14 (0.23) | 1.15 (0.72, 1.81) | 0.56 |
| Intercept | - | -4.10 (1.93) | - | 0.034 |

**Supplementary Table 2.** Final logistic regression model for the association between the presence of quinolone resistance determinants (point mutations and plasmid-mediated quinolone resistance determinants) in *E. coli* genomes and treatment group of calves.

| Factor | Level | Coefficient (SE) | Odds Ratio (95% CI) | P-value |
| --- | --- | --- | --- | --- |
| Treatment group | Zinc | -1.45 (1.16) | 0.23 (0.024, 2.29) | 0.21 |
|  | Placebo | Referent | - | - |
| Days from spectinomycin treatment | - | 0.096 (0.15) | 1.10 (0.81, 1.49) | 0.53 |
| Intercept | - | -2.03 (1.12) | - | 0.070 |

**Supplementary Table 3.** Final logistic regression model for the association between the presence of *aac(3)-VIa* in *E. coli* genomes and treatment group of calves.

| Factor | Level | Coefficient (SE) | Odds Ratio (95% CI) | P-value |
| --- | --- | --- | --- | --- |
| Treatment group | Zinc | 0.79 (0.69) | 2.21 (0.58, 8.50) | 0.25 |
|  | Placebo | Referent | - | - |
| Days from spectinomycin treatment | - | 0.054 (0.11) | 1.06 (0.85, 1.31) | 0.62 |
| Intercept | - | -1.53 (0.82) | - | 0.063 |

**Supplementary Table 4.** Final logistic regression model for the association between the presence of *aadA2* in *E. coli* genomes and treatment group of calves.

| Factor | Level | Coefficient (SE) | Odds Ratio (95% CI) | P-value |
| --- | --- | --- | --- | --- |
| Treatment group | Zinc | 0.48 (0.65) | 1.61 (0.45, 5.72) | 0.46 |
|  | Placebo | Referent | - | - |
| Days from spectinomycin treatment | - | 0.04 (0.10) | 1.04 (0.86, 1.26) | 0.69 |
| Intercept | - | -0.87 (0.73) | - | 0.23 |

**Supplementary Table 5.** Final logistic regression model for the association between the presence of *aadA5* in *E. coli* genomes and treatment group of calves.

| Factor | Level | Coefficient (SE) | Odds Ratio (95% CI) | P-value |
| --- | --- | --- | --- | --- |
| Treatment group | Zinc | -1.04 (0.67) | 0.35 (0.096, 1.30) | 0.12 |
|  | Placebo | Referent | - | - |
| Days from spectinomycin treatment | - | -0.084 (0.11) | 0.92 (0.75, 1.13) | 0.43 |
| Intercept | - | 1.51 (0.81) | - | 0.061 |

**Supplementary Table 6.** Final logistic regression model for the association between the presence of *aph(3'')-Ib* in *E. coli* genomes and treatment group of calves.

| Factor | Level | Coefficient (SE) | Odds Ratio (95% CI) | P-value |
| --- | --- | --- | --- | --- |
| Treatment group | Zinc | 0.84 (0.94) | 2.32 (0.37, 14.71) | 0.37 |
|  | Placebo | Referent | - | - |
| Days from spectinomycin treatment | - | -0.23 (0.18) | 0.80 (0.56, 1.13) | 0.20 |
| Intercept | - | 2.95 (1.38) | - | 0.033 |

**Supplementary Table 7.** Final logistic regression model for the association between the presence of *aph(3')-Ia* in *E. coli* genomes and treatment group of calves.

| Factor | Level | Coefficient (SE) | Odds Ratio (95% CI) | P-value |
| --- | --- | --- | --- | --- |
| Treatment group | Zinc | -0.39 (0.66) | 0.68 (0.19, 2.49) | 0.56 |
|  | Placebo | Referent | - | - |
| Spectinomycin treatment | Yes | -1.30 (1.14) | 0.27 (0.029, 2.54) | 0.25 |
|  | No | Referent | - | - |
| Intercept | - | 1.91 (1.11) | - | 0.084 |

**Supplementary Table 8.** Final logistic regression model for the association between the presence of *aph(6)-Id* in *E. coli* genomes and treatment group of calves.

| Factor | Level | Coefficient (SE) | Odds Ratio (95% CI) | P-value |
| --- | --- | --- | --- | --- |
| Treatment group | Zinc | -0.20 (1.09) | 0.82 (0.096, 7.00) | 0.86 |
|  | Placebo | Referent | - | - |
| Days from spectinomycin treatment | - | -0.29 | 0.75 (0.45, 1.25) | 0.27 |
| Intercept | - | 4.52 (2.17) | - | 0.037 |

**Supplementary Table 9**. Final logistic regression model for the association between the presence of *bla*_CMY-2_ in *E. coli* genomes and treatment group of calves.

| Factor | Level | Coefficient (SE) | Odds Ratio (95% CI) | P-value |
| --- | --- | --- | --- | --- |
| Treatment group | Zinc | -0.23 (1.49) | 0.80 (0.043, 14.65) | 0.88 |
|  | Placebo | Referent | - | - |
| Days from spectinomycin treatment | - | -0.22 (0.33) | 0.80 (0.42, 1.52) | 0.50 |
| Intercept | - | 4.71 (2.65) | - | 0.076 |

**Supplementary Table 10.** Final logistic regression model for the association between the presence of *bla*_TEM-1B_ in *E. coli* genomes and treatment group of calves.

| Factor | Level | Coefficient (SE) | Odds Ratio (95% CI) | P-value |
| --- | --- | --- | --- | --- |
| Treatment group | Zinc | 0.89 (0.69) | 2.44 (0.63, 9.45) | 0.20 |
|  | Placebo | Referent | - | - |
| Days from spectinomycin treatment | - | -0.036 (0.10) | 0.97 (0.79, 1.18) | 0.73 |
| Intercept | - | -1.00 (0.75) | - | 0.18 |

**Supplementary Table 11.** Final logistic regression model for the association between the presence of *dfrA12* in *E. coli* genomes and treatment group of calves.

| Factor | Level | Coefficient (SE) | Odds Ratio (95% CI) | P-value |
| --- | --- | --- | --- | --- |
| Treatment group | Zinc | 0.47 (0.65) | 1.60 (0.45, 5.67) | 0.47 |
|  | Placebo | Referent | - | - |
| Days from spectinomycin treatment | - | 0.049 (0.10) | 1.05 (0.86, 1.28) | 0.62 |
| Intercept | - | -0.93 (0.73) | - | 0.20 |

**Supplementary Table 12.** Final logistic regression model for the association between the presence of *dfrA17* in *E. coli* genomes and treatment group of calves.

| Factor | Level | Coefficient (SE) | Odds Ratio (95% CI) | P-value |
| --- | --- | --- | --- | --- |
| Treatment group | Zinc | -0.81 (0.67) | 0.45 (0.12, 1.64) | 0.22 |
|  | Placebo | Referent | - | - |
| Days from spectinomycin treatment | - | -0.073 (0.11) | 0.93 (0.76, 1.15) | 0.49 |
| Intercept | - | 1.44 (0.80) | - | 0.071 |

**Supplementary Table 13.** Final logistic regression model for the association between presence of *floR* in *E. coli* genomes and treatment group of calves.

| Factor | Level | Coefficient (SE) | Odds Ratio (95% CI) | P-value |
| --- | --- | --- | --- | --- |
| Treatment group | Zinc | -0.44 (0.75) | 0.65 (0.15, 2.78) | 0.56 |
|  | Placebo | Referent | - | - |
| Days from spectinomycin treatment | - | -0.12 (0.13) | 0.88 (0.69, 1.14) | 0.34 |
| Intercept | - | 2.21 (1.00) | - | 0.027 |

**Supplementary Table 14**. Final logistic regression model for the association between presence of *sul1* in *E. coli* genomes and treatment group of calves.

| Factor | Level | Coefficient (SE) | Odds Ratio (95% CI) | P-value |
| --- | --- | --- | --- | --- |
| Treatment group | Zinc | 1.04 (0.67) | 2.83 (0.77, 10.45) | 0.12 |
|  | Placebo | Referent | - | - |
| Days from spectinomycin treatment | - | 0.084 (0.11) | 1.09 (0.88, 1.34) | 0.43 |
| Intercept | - | -1.51 (0.81) | - | 0.061 |

**Supplementary Table 15.** Final logistic regression model for the association between presence of *sul2* in *E. coli* genomes and treatment group of calves.

| Factor | Level | Coefficient (SE) | Odds Ratio (95% CI) | P-value |
| --- | --- | --- | --- | --- |
| Treatment group | Zinc | 0.64 (1.32) | 1.89 (0.14, 25.12) | 0.63 |
|  | Placebo | Referent | - | - |
| Days from spectinomycin treatment | - | -0.35 (0.33) | 0.70 (0.37, 1.34) | 0.28 |
| Intercept | - | 4.98 (2.71) | - | 0.066 |
